# Anatomical Variants of the Origin of the Coronary Arteries: A Systematic Review and Meta-Analysis of Prevalence

**DOI:** 10.3390/diagnostics14131458

**Published:** 2024-07-08

**Authors:** Juan José Valenzuela Fuenzalida, Emelyn Sofia Becerra-Rodriguez, Alonso Sebastián Quivira Muñoz, Belén Baez Flores, Catalina Escalona Manzo, Mathias Orellana-Donoso, Pablo Nova-Baeza, Alejandra Suazo-Santibañez, Alejandro Bruna-Mejias, Juan Sanchis-Gimeno, Héctor Gutiérrez-Espinoza, Guinevere Granite

**Affiliations:** 1 Departamento de Morfología, Facultad de Medicina, Universidad Andrés Bello, Santiago 8370146, Chile; juan.kine.2015@gmail.com (J.J.V.F.); alonsoquivira@gmail.com (A.S.Q.M.); belenbaezflores@gmail.com (B.B.F.); e.catalina.n21@gmail.com (C.E.M.); pablo.nova@usach.cl (P.N.-B.); 2 Departamento de Ciencias Química y Biológicas, Facultad de Ciencias de la Salud, Universidad Bernardo O’Higgins, Santiago 8370993, Chile; 3 Escuela de Medicina, Unidad Central del Valle del Cauca, Tuluá 763022, Colombia; emelyn.becerra01@uceva.edu.co; 4 Escuela de Medicina, Universidad Finis Terrae, Santiago 7501015, Chile; miorellanadonoso@gmail.com; 5 Faculty of Health and Social Sciences, Universidad de Las Américas, Santiago 8370040, Chile; alej.suazo@gmail.com; 6 Departamento de Ciencias y Geografía, Facultad de Ciencias Naturales y Exactas, Universidad de Playa Ancha, Valparaíso 2360072, Chile; alejandro.bruna@unab.cl; 7 GIAVAL Research Group, Department of Anatomy and Human Embryology, Faculty of Medicine, University of Valencia, 46001 Valencia, Spain; juan.sanchis@uv.es; 8 One Health Research Group, Universidad de las Américas, Quito 170124, Ecuador; 9 Department of Surgery, Uniformed Services University of the Health Sciences, Bethesda, MD 20814, USA; guinevere.granite@usuhs.edu

**Keywords:** coronary artery, left coronary artery (LCA), right coronary artery (RCA), origin anomalous coronary artery, clinical anatomy, anatomical variation

## Abstract

Purpose: The most common anomaly is an anomalous left coronary artery originating from the pulmonary artery. These variants can be different and depend on the location as well as how they present themselves in their anatomical distribution and their symptomatological relationship. For these reasons, this review aims to identify the variants of the coronary artery and how they are associated with different clinical conditions. Methods: The databases Medline, Scopus, Web of Science, Google Scholar, CINAHL, and LILACS were researched until January 2024. Two authors independently performed the search, study selection, and data extraction. Methodological quality was evaluated using an assurance tool for anatomical studies (AQUA). Pooled prevalence was estimated using a random effects model. Results: A total of 39 studies met the established selection criteria. In this study, 21 articles with a total of 578,868 subjects were included in the meta-analysis. The coronary artery origin variant was 1% (CI = 0.8–1.2%). For this third sample, the funnel plot graph showed an important asymmetry, with a *p*-value of 0.162, which is directly associated with this asymmetry. Conclusions: It is recommended that patients whose diagnosis was made incidentally and in the absence of symptoms undergo periodic controls to prevent future complications, including death. Finally, we believe that further studies could improve the anatomical, embryological, and physiological understanding of this variant in the heart.

## 1. Introduction

The heart is supplied primarily by two branches of the ascending aorta, the left coronary artery (LCA) and the right coronary artery (RCA). As its name suggests, the LCA is responsible for supplying the left side of the heart, starting its journey from the left coronary sinus (LCS) or the left sinus of Valsalva (LSV) and then dividing into the anterior interventricular artery or left anterior descending artery (LAD) and the left circumflex artery (LCx). On the contrary, the RCA begins its journey through the right coronary sulcus (RCS) or the right sinus of Valsalva (RSV), passing to the anterior atrioventricular sulcus to then anastomoses with the LCx, thus supplying the right side of the heart. Although the pathways described are most common for the LCA and RCA, anomalous or different origins can occur, as well as changes in their destination, location, or shape [1,2].

Different types of coronary anomalies can be described and classified according to their origin, course, and termination, or by hemodynamic alterations. However, these anomalies are caused by an alteration at the embryonic level (congenital defect) during the early development of the coronary arteries. In these cases, proper growth of the fetus can be affected or cause sudden death. The most common anomaly is an anomalous LCA originating from the pulmonary artery (PA), which is characterized by the origin of the LCA in the PA instead of the LCS. This syndrome is more common in infants and often presents with accompanying symptoms. At the same time, it is less common in adults and is usually asymptomatic. Other existing variants are abnormalities in the LCx, abnormalities in the RCA, LCA, the left main coronary artery (LMCA), and single coronary artery, among others [1,3,4].

Although there are various diagnostic methods for the detection of coronary anomalies, the use of computed tomography (CT) currently prevails, because this type of imaging indicates the arterial path from its origin to its destination with good resolution and without being invasive for the patient. Another effective but less frequent method is coronary angiography, which gives us a three-dimensional resolution of the arterial pathway, although this is more invasive and has a high economic value, which makes it less accessible [5,6,7]. The literature describes the prevalence of coronary artery anomalies as rare (0.21–5.79%). Due to the greater sensitivity of tomographic studies, these anomalies are more commonly observed using these imaging methods compared to conventional angiography [8,9].

Although the precise reason why the anomaly occurs is unknown, it carries several risks for symptomatic people. If a coronary artery is affected, blood flow in the myocardium can be hindered and can cause myocardial ischemia, and in the worst cases, sudden death. The most frequent symptoms can be fainting during physical activity, respiratory problems, heart problems, and chest pains, which is why people who suffer from this disease and practice intense physical activities are more likely to die from this anomaly [10,11,12,13].

This review aimed to determine the characteristics of coronary artery variants and their relationship with cardiac clinical considerations.

## 2. Methods

### 2.1. Protocol and Registration

To carry out this meta-analysis, we were guided by the Prisma statement [14]. The registration number in the Systematic Reviews Registry (PROSPERO) is CRD42024520734.

### 2.2. Electronic Search

To obtain the best studies that fit our research question, we searched the following databases in January: MEDLINE (via PubMed), Google Scholar, Web of Science (WOS), Cumulative Index to Nursing and Allied Health Literature (CINAHL), Latin American and the Caribbean Literature in Health Sciences (LILACS), and Scopus from their inception until March 2024. Our Search strategy included a combination of the following terms: “coronary artery” (Not Mesh), “left coronary artery” (Not Mesh), “right coronary artery (Not Mesh), “origin anomalous coronary artery” (Not Mesh), “clinical anatomy” (Not Mesh) and “anatomical variation”(Not Mesh), using the Boolean connectors AND, OR, and NOT (Supplementary Table S1).

### 2.3. Eligibility Criteria

As eligibility criteria, studies that included the presence of origin coronary artery (OCA) variants and their associations with some clinical conditions were included. They were considered eligible for inclusion if the following criteria were met: (1) Sample: dissections or images with the presence of the OCA variation; (2) Results: prevalence of subjects who presented OCA variants and their correlation with pathologies of the cardiac region; (3) Studies: This systematic review included research articles, retrospective and prospective observational types, published in English in peer-reviewed journals and indexed in the reviewed databases.

As exclusion criteria, we used the following to eliminate studies from our selection: (1) sample: studies carried out in animals; (2) studies that analyzed variants of the region or system outside the hepatic region or its drainage area or tract; (3) studies including letters to the editor or comments.

### 2.4. Study Selection

To make a thorough selection of the studies, we analyzed three authors independently. In the first instance, two authors (Valenzuela JJ and Escalona C) examined the titles and abstracts of the references recovered from the database searches. For the selected studies, the full texts of the references that any of the authors considered potentially relevant were obtained. A third reviewer (Quivira A) was involved if a consensus could not be reached. For this purpose, we also performed the agreement test between authors, the kappa test, to analyze reliability and the risk of bias between observers, which in this case gave 0.80, which is interpreted as good agreement.

### 2.5. Data Collection Process

Two authors (Nova P and Orellana M) independently extracted data on the outcomes of each study. The following data were extracted from the included studies: (a) authors and year of publication, (b) example total *n* and age, (c) prevalence, (d) characteristics of variant, (e) region geography, (f) sex of the sample, and (g) clinical considerations.

### 2.6. Assessment of the Methodological Quality of the Included Studies

To evaluate the bias of the included studies, we used the verification table for anatomical studies (AQUA) proposed by the International Working Group on Evidence-Based Anatomy (IEBA) [15]. Two reviewers (Valenzuela JJ and Nova P) independently analyzed the five domains proposed by the AQUA tool, reached a consensus and constructed the table and bias graph.

### 2.7. Statistical Methods

The data extracted from the meta-analysis were interpreted by calculating the VAH prevalence using JAMOVI software 2.1212 (https://www.jamovi.org/ accessed on 4 January 2024). The DerSimonian–Laird model with a Freeman–Tukey double arcsine transformation was used to combine the summary data. In addition, a random effects model was used because the VAH prevalence data were highly heterogeneous. The degree of heterogeneity between the included studies was assessed using the chi^2^ test and the heterogeneity (I^2^) statistic. For the chi^2^ test, the *p*-value proposed by the Cochrane collaboration was considered significant at 0.10. Values of the I^2^ statistic were interpreted with a 95% confidence interval (CI) in the following way: 0–40% might not be important, 30–60% might indicate moderate heterogeneity, 50–90% might represent substantial heterogeneity, and 75–100% could represent a significant amount of heterogeneity.

## 3. Results

### 3.1. Selection of Articles

The search process yielded a total of 795 articles from various databases, aligning with the criteria and search terms established by our research team. A filtration process was applied, focusing on the titles and/or abstracts of these articles. Out of the initial pool of 70 articles included, 39 articles were selected for inclusion in the meta-analysis. These articles were chosen based on their comprehensive study of the sample, detailed statistical data for each variant, and their utilization of a clear methodology.

Conversely, eight articles, primarily consisting of clinical case reports, were excluded from the meta-analysis. These reports, while offering valuable clinical and anatomical insights, primarily detailed individual cases and thus lacked the broader statistical foundations required for substantive analysis of the variables. However, these case reports were still considered valuable for the clinical and anatomical aspects of this study.

**Figure 1:**
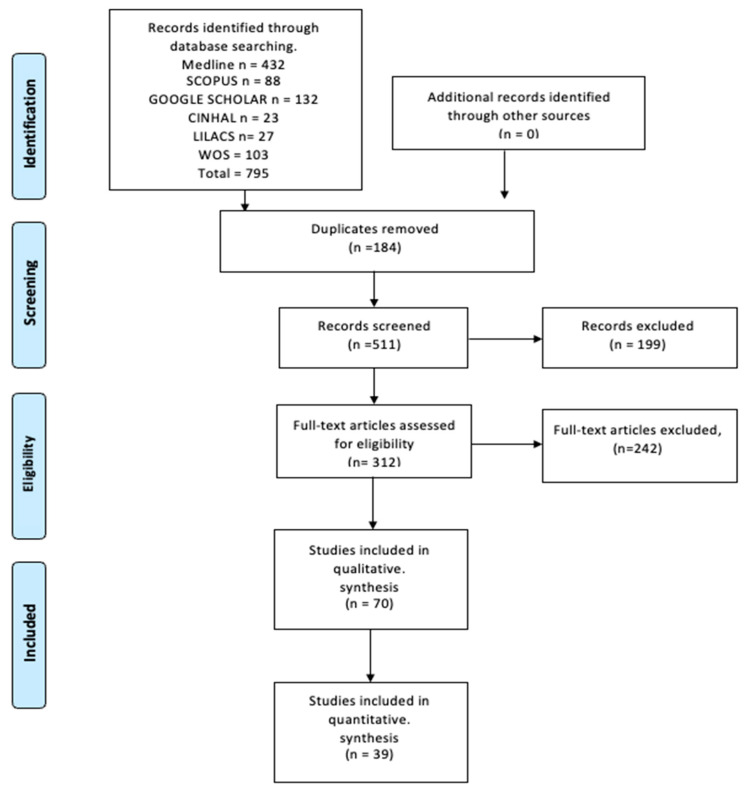
Flow diagram of the included studies.

The total sample size encompassed 54,588 individuals. For the meta-analysis, 51,244 participants, derived from the 39 selected articles, were included. This sample comprised patients, imaging studies, and donor specimens (Figure 1).

### 3.2. Characteristics of Included Studies

A total of 70 studies that met our inclusion criteria were included in this review; they had a cumulative N of 578,868 subjects. Within these 70 studies, 38 met the criteria for the prevalence meta-analysis [2,5,6,7,10,12,13,16,17,18,19,20,21,22,23,24,25,26,27,28,29,30,31,32,33,34,35,36,37,38,39,40,41,42,43,44,45,46,47,48,49,50,51,52,53,54,55,56,57,58,59,60,61,62,63,64,65,66,67,68,69,70,71,72]. Conversely, according to the variants reported by the studies, three reported variants in the origin of the branches of the RCA, 17 reported variants in the origin of the branches of the LCA, and 43 reported variants directly from the coronary artery. However, only 34 subjects out of 7400 reported anatomical variants in the LCA, while only 28 out of 1750 subjects reported variants in the RCA.

Geographically, 34 of the studies came from Asia, 2 from the African continent, 2 from South America, 14 from North America, and 14 from the European continent. In addition, the average age of the subjects studied was 28 years, with a standard deviation of 24 years. There were 8048 male subjects and 5723 female subjects, while 565,097 subjects had no reported sex (Table 1).

### 3.3. Description of Variants

**Figure 2:**
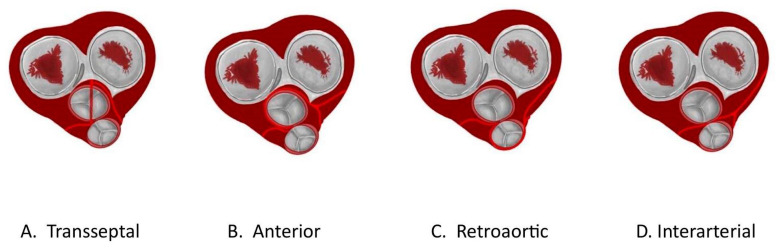
Different origins of coronary arteries. (**A**) shows a trans-septal course of the AC indicated in light red, while (**B**) shows the course of the AC anteriorly. (**C**) exemplifies a route of the retroaortic AC, whereas (**D**) shows an interatrial route of the AC.

When discussing anomalies of origin, the affected segment at the beginning of the coronary artery is examined. It can have a high or low origin, with an incidence of 6%. The coronary ostium, located at the lower end of the coronary sinus, is called the low origin, and the coronary ostium located 5 mm from the valvular attachment point of the aortic annulus is called the coronary commissure ostium. A high-origin ostium refers to an origin more than 10 mm away from the sinotubular junction. It is more common for the right coronary artery to have a high ostium of origin, which can be benign but hemodynamically significant. Likewise, multiple ostia are present in 0.41–0.43% of the population and present as ostia separated from the LCx and the LAD [40]. The single coronary artery, on the other hand, comes from the aortic sinus. It is usually benign, follows the course of the LAD or RCA, and may divide into two or three main branches after its onset. If there is an interarterial pathway between the PA and the aorta, the risk may increase. In coronary artery anomalies of PA origin, the most common form is the left coronary artery anomaly of the PA (ALCAPA), in which the left principal coronary arteries (TCI) arise from the PA. It occurs in 1 in 300,000 live births, and 90% will die within the first year of life if left untreated. Another abnormality is the RCA arising from the PA (ARCAPA); here, the ostium of the RCA arises from the PA. It is rarer and usually asymptomatic. There is also atresia or congenital stenosis of the ostium, which is rare and corresponds to a congenital malformation of the coronary artery, in which there is partial or complete occlusion of the RCA or LCA, which has been associated with hypoplasia of the proximal segment of the corresponding coronary artery (Figure 2).

**Figure 3:**
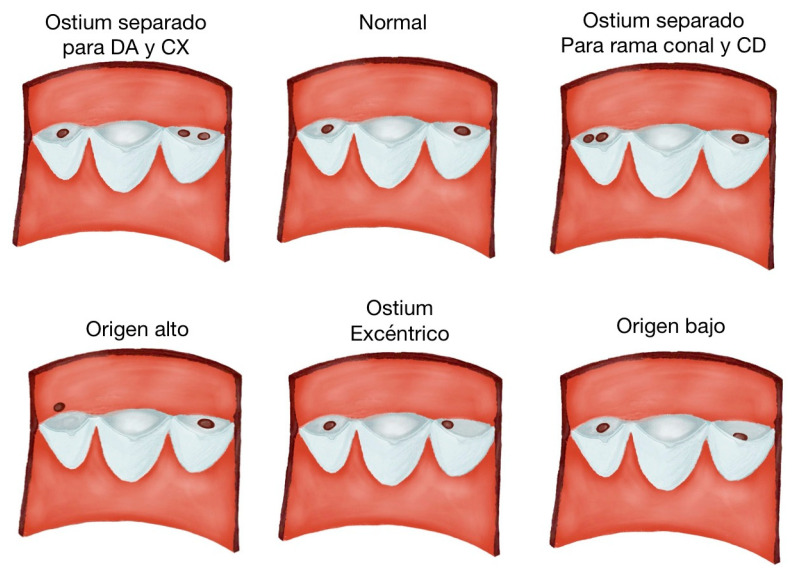
Variations in the origins of coronary arteries. The image shows the different origins of the coronary arteries in which positions vary, whether from a high or low origin, a double origin in the different valves, or a more eccentric origin.

On the other hand, there are also anomalies regarding the origin of a coronary artery or one of its branches in the opposite sinus or the non-coronary sinus. These anomalies can present in four types. The intra-arterial, which occurs between the pulmonary artery and the aorta, is the most important clinically since it is the most common cause of sudden death in athletes. The other three courses are retroaortic, prepulmonary, and transseptal, all of which are benign. Regarding course abnormalities, we recognize the intramyocardial course (myocardial bridge) in which the coronary artery tunnels into the heart muscle. In most cases, it is a benign and asymptomatic variant. It is described as complete if part of the coronary artery is lined by muscle bundles, or incomplete if only one layer of fibres covers the coronary artery. The different types of bridges are right ventricle, deep septal, and superficial bridges, which can be complete or incomplete (Figure 3).

Another anomaly is the duplication of the LAD and the divided RCA, which is a benign variation, where the RCA arises from two ostia or arises from one ostium and divides into two arteries after its origin. Split LCA is extremely rare, so the incidence of LAD duplication is 1% and has been documented only in sporadic cases. There are four types of duplications depending on the trajectory. There is the long distal anterior descending artery that runs along the left side of the interventricular sulcus, the long distal ADA that runs on the right side of the interventricular sulcus, the long distal ADA that has an intramyocardial course in the septum and appears on the epicardial surface in the distal part of the interventricular sulcus, and the long distal ADA that originates from the coronary artery (CA). Finally, coronary ectasia or aneurysm is defined as an enlargement or dilation of at least 1.5 times its normal diameter. If the entire blood vessel is affected, it is called ectasia, while if it is only partially affected, it is called a coronary aneurysm [20]. Congenital symptoms are very rare and commonly occur as a result of Kawasaki disease or a coronary artery fistula.

Regarding termination anomalies, we found three types: Coronary arterial fistula, which treats the abnormal termination of a coronary artery in the ventricle, the cardiac vein, or the PA [27]. The RCA is affected in 44% of cases, the left main coronary artery (LAB) in an additional 44%, and both TCI and RCA in 12% of cases [61]. Then there is the extracardiac or systemic termination, which is a termination anomaly that must be distinguished from a coronary fistula. This is because, in the fistula, the coronary artery is dilated and tortuous, unlike the systemic endings that do not have this pattern since there is no significant pressure difference between the coronary artery and the systemic artery to which it empties. Finally, the coronary arch is a direct connection between two main coronary arteries without the need for occlusive injury. Anatomical continuity has been reported, especially between the LCx and the RCA.

### 3.4. Prevalence and Risk of Bias

**Figure 4:**
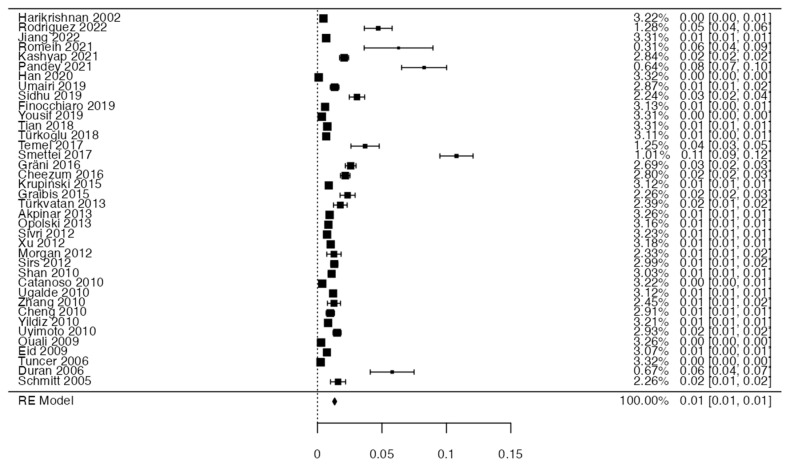
Forest plot of prevalence of variants of the origin of the coronary arteries.

**Figure 5:**
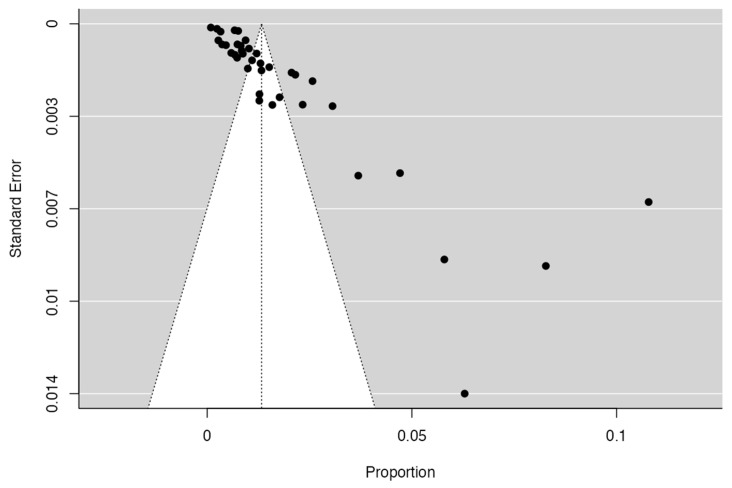
Funnel plot of prevalence of variants of the origin of the coronary arteries.

To calculate the prevalence of OCA variants in the studies included in this review (Table 2), one proportion of forest plots was made. To include the studies in the prevalence forest plot it was taken into account that the studies had to have reported a proportion of at least 10%. A total of 38 studies that met the criteria were included for variants of the OCA [2,4,6,11,12,13,19,20,21,22,26,27,28,33,34,37,45,49,51,55,56,57,62,63,64,65,66,67,68,69,73,74,75,76,77,78,79]. The Forest plot diagram showed that the prevalence was 1%, with a confidence interval of 0.8–1.2% (Figure 4 and Table 2). For this third sample, the funnel plot graph showed an important asymmetry, which had a *p*-value of 0.162 and is directly associated with this asymmetry (Figure 5).

**Figure 6:**
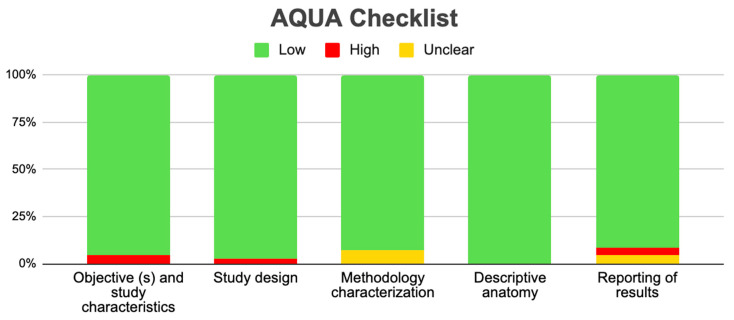
The risk of bias in studies included, as determined using the AQUA checklist tool.

A total of 70 studies met the criteria to be evaluated using the AQUA Checklist for anatomical studies tool in which bias was analyzed in five domains. In the five domains offered by the AQUA Table, the three included studies presented a low risk of bias and were analyzed across all the domains (Figure 6).

### 3.5. Clinical Considerations

Several theories attempt to explain the mechanisms that generate cardiac ischemia. In sudden cardiac death, it has been proposed that the mechanism of ischemia is the production of a transient or sustained spasm in the muscular layer of an anomalous coronary artery since sudden cardiac death is the main manifestation of the variation of the coronary arteries or their origin. This has been attributed to the endothelial damage of the blood vessel, which in turn has been directly associated with the abnormal trajectory and OCA. Finally, other authors showed that an intussusception of the proximal part of the CA into the aortic wall occurs. This anomaly only gives symptoms in 20% of patients since in the other patients it can be present without producing any type of damage or symptomatology. If the symptoms are present, they will manifest as dyspnea with associated syncope or some type of angina such as exertional angina. Another theory that explains the signs and symptoms in variants of AC is that they appear when an angulated location is presented by the anomalous AC in their ascending aortic artery exit. The difference with a normal AC is that the AC with variant has a location more perpendicular to the aorta, while the abnormal AC must be adapted so that it “flexes” on itself to reach from the sinus of the contralateral leaflet to its irrigation territory [19,38,46,61,71]. The clinical presentation is variable, ranging from asymptomatic patients to patients with angina, dyspnea, and syncope, either at rest or with exercise, acute myocardial infarction, heart failure, and sudden death. It has been observed that when symptoms are present in patients under 30–35 years of age there is an increased risk of sudden death, and in up to 40% of cases, symptoms that manifest during or immediately after physical activity precede death [16,28,35,39,46,53,62,71,78,79,80,81]. As they are mostly asymptomatic, the diagnostic reference standard is coronary angiography, and more imaging findings are made incidentally when performing imaging studies for other causes [25,46,73,79,81]. Coronary anomalies occur in 1.7% of the general population and cause sudden deaths in 33% of the young population during strenuous effort; the use of imaging techniques to diagnose these variants has grown [64,71,73]. Coronary artery anomalies can be classified according to Greenberg as anomalies of origin, course, and termination. However, one of the factors that most influence the classification is whether it has a hemodynamic impact.

## 4. Discussion

It was found that most of the subjects under study came from an Asian lineage, so there may be a predominance of certain anomalies based on nationality; however, there are no studies that support this association. If we talk about the age of the subjects in whom coronary anomalies were studied, the average age was 28 years, which means that this condition is detected mostly in adult subjects. In this study, we cannot infer whether these anomalies are related to or are more present in male or female subjects since the subjects’ reports did not place much importance on sex. Regarding the variants most present in this study, it was possible to visualize an abundance of origin-type anomalies, i.e., cases of anomalous origins of the coronary artery are more frequent than anomalies in its route or termination.

Regarding previous studies that have analyzed the variants of the origin of AC, our search only found three studies that met the criteria similar to the objectives set out in this review. The first study, and the one that presents the greatest similarity to ours, is that of Ponzoni et al. 2022 [81], which showed in its results the outcome of surgeries in variants of anomalous aortic origin of coronary arteries in children and young adults (<30 years). Thirteen publications, including a total of 384 patients, were selected. Surgical treatment of the anomalous aortic origin of the coronary arteries can be achieved with excellent results in pediatric patients, but concerns persist about the durability of the surgery, which could be a trigger for future pathologies. In addition, we conducted an exhaustive study of the anatomical considerations of anomalous OCA. Finally, we associated all the above with important clinical considerations that must be taken into account in the presence of this variant. On the other hand, the D’Ascenzi, 2022 [79] study, although it did not show the anatomy of AC, states that deaths from heart attacks could be associated with variants in the origin of AC. Although these characteristics are associated with the clinic, this study does not detail the anatomy or how the variant can produce cardiac pathologies. Finally, the Koppel, 2020 [35] review found the prevalence of coronary anomalies in tetralogy of Fallot to be 4–6%. In patients with an abnormal coronary artery, 72% cross the abnormal outflow tract of the right ventricle. The combined risk of finding an anomalous coronary artery or a large cone artery crossing the right ventricle is 10.3%. The coronary anatomy should be defined before surgery and the surgical approach should be adapted if necessary. Although this review makes a detailed analysis of the anomalous origin of AC in the presence of a tetralogy of Fallot, our study analyzes all the pathologies reported in studies with variants and the number of our studies and subjects is greater, which is why our study is novel.

Both the number of studies and the number of subjects investigated were considerable—more than 550,000 accumulated from all the studies—and shows that this anatomical variant is frequently studied and reported in the literature. If we focus on the data obtained in this study, most came from Asia, which could lead us to think that this variant is associated with races from the Asian continent. However, we have not found any study that supports this theory, so we believe that the greater number of studies in this continent is associated with the availability of samples and studies carried out in that section of the world. Regarding the characteristics of the sex of the sample, we cannot make a direct relationship between this variable and the presence of variation in the origin of the CA. This is because most of the studies included in this review did not indicate the sex of the subjects, with more than 500,000 subjects not reporting the sex of the subjects. The sex of 13,771 subjects was reported, which is equivalent to only 2% of the total sample. Among these subjects, there were 8048 men and 5723 women; therefore, we are categorical that for this study a relationship between sex and the presence of variants in the origin of AC cannot be attributed. On the other hand, according to the variants reported, most of the studies presented direct variants of the OCA. In addition, most of the cases were bilateral, that is, this anomaly occurred in both the LCA and the RCA. Another point is the average age of the subjects studied, which was 28 years, highlighting the early age of recognition of this variant since it causes symptoms in many subjects and great attention must be paid to the presence of variants in the origin of AC.

In the heart, the coronary arteries arise from the aortic wall from a plexus or peritoneal ring that connects to the systemic circulation; however, when this does not occur as it should, an anomaly, which does not have a clear definition, is formed. Despite this, a pattern has been used to classify variants found in the coronary tree; these are based on clinical significance or anatomical and functional characteristics. They are anomalies of origin, which in turn are divided into high and low origin, multiple ostia, single coronary artery, anomalous origin of coronary artery in the pulmonary artery, origin of a coronary artery or one of its branches in the opposite sinus or the non-coronary sinus, with abnormal course, atresia, or congenital stenosis of the ostium; course anomalies that in turn are divided into atresia or congenital stenosis of the ostium, duplication of LAD and divided RCA, ectasia or coronary aneurysm; and termination anomalies that include coronary arterial fistula, extracardiac or systemic termination, and coronary arch.

Anomalies of the coronary arteries are rare in the general population; however, the most frequently found are anomalies of origin and those of the trajectory of the central segments, that is, one is born with one of the coronary arteries from the opposite coronary sinus. The most common variant was the ectopic origin of the RCA from the LSV or the proximal LCA, and the main route was the intra-arterial route (Harikrishnan et al., 2002; Schmitt et al. 2005; Eid et al. 2009; Yildiz et al. 2010 [13,29,53,70]). The prevalence of the variants in the origin of AC was very low; cumulatively, it was less than 1%, indicating that this variant occurs in isolation. In this study, we did not separate the prevalence by different variants since if we had conducted this exclusion, the samples would have been very heterogeneous and limited. Measuring the bias of inclusion in the meta-analysis of the studies using a funnel plot showed symmetry in most of the included studies; thus, it can be said that the statistical analysis was relevant. On the other hand, the bias in the methodology was measured using the AQUA tool.

Because the coronary arteries are responsible for transporting oxygenated blood to the heart muscle, any morpho-functional alteration could cause reduced oxygen and nutrient transport to the heart. We have seen that defects in the origin of the coronary arteries can be multiple, but those closely related to the origin of heart irrigation can cause the most alterations. Therefore, anomalies in the RCA and LCA could potentially cause the most significant alterations. A coronary artery defect can present with a wide spectrum of clinical manifestations. Due to the various types of anomalies that can occur at the origin of the coronary arteries, some patients may be asymptomatic, while others may experience atypical anginal chest pain, syncope, dyspnea, angina, heart failure, acute heart attack, or even sudden death in severe cases. Symptoms have been mostly associated with young adults than the elderly. Additionally, cases of death, although occurring in adults, often happen at an early age, as reported in the literature, and are often associated with syncope at an early age. This could be linked to dietary alterations, lifestyles involving alcohol and drugs, and infrequent medical check-ups. Therefore, early diagnosis suggests periodic follow-up of these patients.

## 5. Limitations

This review was limited by the publication and authorship bias of the included studies. First, studies with different results that were in non-indexed literature in the selected databases may have been excluded. Second, there could be limitations in the sensitivity and specificity of the searches. Finally, the authors selected articles personally. All of these increase the probability of excluding potential cases from countries outside of Asia and North America that are not reported in the scientific community.

## 6. Conclusions

An anomalous origin of the variants of AC is a variant that has a low prevalence but has been exhaustively studied in the scientific literature. In this study, we found that the presence of this variant in AC could cause symptoms of high clinical significance, reaching the point of being a cause of death. For this reason, we believe that the knowledge of cardiac surgeons is crucial in avoiding functional and irrigation alterations. It is recommended that patients whose diagnosis was made incidentally and in the absence of symptoms undergo periodic controls to prevent future complications, including death. Finally, we believe that further studies could improve the anatomical, embryological, and physiological understanding of this variant in the heart.

## Figures and Tables

**Table 1:** Characteristics of the studies included in the review.

Author	N of Subjects and Diagnostic Methods	Variant	Prevalence	Age	Location	Male or Female	Clinical Considerations
Harikrishnan et al., 2002	7400 coronary angiography	Coronary anomalies	34 cases. The most common anomaly was the origin separation of the left anterior descending artery (LAD) and left circumflex artery (LCx)	50	Does not report	22 men and 12 women	Does not report
Michielon et al., 2003	31 electrocardiogram and echocardiogram	Origin anomalous of the coronary artery from the PA	31 anomalous origin left coronary arteries (*n* = 28), right (*n* = 2), both (*n* = 1) of pulmonary artery (PA)	Range, 1.1–203 months	Rome	16 men and 15 women	Does not report
Schmitt et al., 2005	1758 TC multidetector (TCMD)	Congenital anomalies of coronary arteries	28 were found, where 12 anomalies were not hemodynamic and affected only coronary origins (*n* = 10) or the path of peripheral vessels.	37–83	Germany	Does not report	Chest pain
Durán et al., 2006	725 coronary angiography	Anatomical anomalies of coronary arteries	42 ectopic origin of the LAD (*n* = 1, 0.13%), absence of left coronary artery (LCA), principal coronary artery (*n* = 4, 0.52%)	33–78	Afghanistán	497 patients with 228 women	Chest pain
Tuncer et al., 2006	70,850 coronary angiography	Origin anomalies and distribution of LAD	171	18–84	Turkey	99 men and 72 women	Does not report
Osaki et al., 2008	35 donors	Origin anomalies of the coronary artery opposite to the sinus of Valsalva.	35 were found. 13 left principal coronary arteries (TCI) intra-arterial (42%) and 18 right coronary arteries (RCA) intra-arterial (58%)	From Newborn to 16 years old	Canada	25 men and 6 women	Does not report
Eid et al., 2009	4650 angiographies	Anomalous aortic origin of the coronary arteries	34 were found. The anomalous LCx was the most common (19 of 34 patients), while the second most common abnormality was the anomalous origin of coronary right (CR) (9 of 34 patients)	30–85 years old	Lebanon	26 men and 8 women	Chest pain, palpitations, and dyspnea or exertional angina
Ben Ali et al., 2009	62 surgery	LCA from the PA	62 boys with coronary artery (CA) from PA	10 days–11 years old	France	Does not report	Congestive heart failure
Ouali et al., 2009	7330 diagnostic coronary angiography patients	Coronary artery anomaly	20 were found. The RCA was the most frequently affected vessel (*n* = 10). The isolated anomalous CX was the second most common anomaly (*n* = 6).	21–72 years old	Túnez	13 men and 7 women	Myocardial infarction
Fujimoto et al., 2010	5869 multidetector computed tomography	Coronary artery anomaly origin	89	65.9	Japan	3.186 men and 2.683 women	Associated with sudden death and ischemic heart disease
Yildiz et al., 2010	12,457 coronary angiography	Coronary artery abnormalities	112 were found. The most common anomaly was the separation of the origins of the LAD and LCx arteries from the left sinus of Valsalva (LSV) (63.4%).	22–79 years	Turkey	70 men and 42 women	Arrhythmias, syncope, myocardial infarction, or sudden death
Zheng et al., 2010	23 echocardiogram, angiography, computed tomography (CT), or confirmed during cardiac surgery.	Anomalous origin of the LCA from the PA (ALCAPA)	23	2.5 months–65 years old	China	13 men and 10 women	Does not report
Cheng et al., 2010	3625 coronary angiography DSC	Coronary artery abnormalíties	36 were found; 11 anomalies of the RCA; 5 anomalies of the LCA; 10 anomalies of the LCx; 2 single coronary artery	15–76 years old	China	20 men and 16 women	Chest pain during exercise
Zhang et al., 2010	1879 angiography for TC, dual source	Anomalous origin of the coronary artery (OCA)	24 were found; 15 patients had an anomalous origin of artery CR (12 of the breast CI, 3 high takeoff), and 8 patients had an anomalous origin of artery coronary left (CL).	25–91 years old	China	1017 men and 864 women	Chest pain during exercise or difficulty breathing
Ugalde et al., 2010	10,000 coronary angiography	Anomalous OCA	121 were found. The most common anomaly was the origin of the RCA from the left coronary sinus (LCS) in 75%, followed by the origin of the right circumflex artery (RCx) in 20%.	58 years old	Chili	70% men	Does not report
Catanoso et al., 2010	6300 coronary angiography	Coronary artery anomalous	23	35–79 years old	Rome	20 men and 3 women	Angina and ventricular arrhythmias
Yang et al., 2010	6014 computed tomography	coronary artery abnormalities	66	4–82 years old	China	5 men and 21 women	Chest pain, dyspnea, palpitations, myocardial infarction, or various arrhythmias.
Mainwaring et al., 2011	50 surgical repair	Anomalous aortic OCAs	50 were found; 31 had the right coronary artery originating in the LSV, 17 had the LCA originating in the right sinus, and 2 had a single eccentric coronary ostium.	5 days–7 days	USA	36 men and 14 women	Chest pain, syncope or near syncope, myocardial infarction, or heart failure
Rahman et al., 2012	24 patients	Anomalous aortic OCAs	21		Bangladesh	Doe not specify	Associated with angina pectoris, arrhythmias, sudden death, myocardial infarction, syncope, and congestive heart failure
Sohrabi et al., 2020	6065 catheterization cardiac	Abnormalities in the coronary arteries	79 were found. The most common abnormality was separate ostia of the LAD and the LCx, which was found in 42 patients (53.16%).	34–84 years old	Irán	58 men and 21 women	Chest pain
Morgan et al., 2012	1570 Percutaneous intervention	Anomalous circumflex coronary arteries	20 were found. In 9 cases the circumflex arose from the left coronary cusp, in 7 cases it arose from the right coronary cusp, and in the remaining 4 cases it arose from the proximal RCA	Does not report	England	Does not report	Chest pain, ECG changes, and troponin elevation
Xu et al., 2012	12,145 DSCT-CA dual-source computed tomography coronary angiography	Coronary artery anomalies	124. An anomalous origin of the LCx from the right sinus of Valsalva (RSV) or the RCA was described in 17 patients. An anomalous origin of the left main artery from the RSV was described in 1 patient.	5–86 years old	China	80 men and 44 women	Chest pain, syncope, or dyspnea on exertion
Sivri et al., 2012	12,844 coronary angiography	Coronary artery anomalous	95 were found. The LCx of the RSV or the RCA are the most prevalent (46 of 95 patients), and the second most common anomaly is the anomalous aortic origin of the RCA (32 of 95 patients).	Does not report	Tukey	69 men and 26 women	Does not report
Opolski et al., 2013	8522 coronary computed tomography angiography	Coronary anomalies originating from the opposite sinus of Valsalva	72 were found. Right-sided origins of the LCA (*n* = 11), the LAD (*n* = 9), the LCx (*n* = 33), and the left origin of the RCA (*n* = 20).	12–93 years old	Poland	37 men and 35 women	Atypical chest pain and anginal pain
Akpinar et al., 2013	25,368 coronary angiography	Coronary anomalies	238 were found. The most common was a LAD circumflex artery originating from the separated ostium (0.29%). The second most common anomaly was an RCA originating from the left sinus of Valsalva (LSV) (0.23%)	30–82 years old	Turkey	92 women and 14 men	Coronary pain, angina, and chest pain
Türkvatan et al., 2013	2375 multidetector computed tomography	Coronary arteries anomalies	42	18–82 years old	Turkey	24 men and 18 women	Chest pain
Mainwaring et al., 2014	76 stress tests, stress echocardiography, and myocardial perfusion studies	Anomalous aortic OCA	65 were found; 38 were anomalous RCAs and 17 were anomalous LCAs	15–47 years old	USA	55 men and 21 women	Chest pain and syncope
Muzaffar et al., 2014	53	ALCAPA	53	4 months	India	29 men and 24 women	Does not report
Kudumula et al., 2014	25	ALCAPA	25	Does not report	England	7 men and 18 women	Arrhythmias, chest pain, heart failure, and sudden death.
Graidis et al., 2015	2572 64-slice MDCT coronary angiography	Congenital coronary anomalies	60 were found. In 16 patients, an anomaly in the left main coronary artery (LMCA) and both in 2 patients. A separate origin of the (LAD and CX) from the (LSV) was found in 15 patients. In 9 patients the RCA arose from the opposite sinus of Valsalva with a separate ostium. An abnormal origin of LCx was found in 6 patients.	29–80 years old	Greece	83.3% men	Chest pain
Angelini et al., 2015	67 baseline intravascular ultrasound	Origin of the RCA from the opposite sinus of Valsalva	67	12–73 years old	USA	67% men	Chest pain and dyspnea
Mongé et al., 2015	36 transthoracic echocardiography (TTE) and cardiac catheterization	Anomalous origin of the LCA from the PA	36 were found. The anomalous coronary artery arose from the main PA in 3 patients, the right PA in 2, and from the junction of the right and main PAs in 2.	From 14 months to 18 years old	USA	18 men and 18 women	Does not report
Krupiński et al., 2015	7115 patients undergoing cardiac CT	Anomalous OCA	62 were found. Anomalous aortic and pulmonary OCA affected 59 (0.83%) and 3 (0.04%) cases, respectively.	17–80 years old	Poland	34 men	Does not report
Gräni et al., 2016	5634 coronary computed tomography angiography (CCTA)	Anomalies of the coronary artery	145 were found; 49 patients showed malignant CAAs, and the remaining 96 patients (66.2%) were classified as having benign variants.	Does not report	Switzerland	Does not report	Does not report
Agrawal et al., 2016	61	Anomalous aortic OCA	53	8–18 years old	USA	Does not report	Does not report
Cheezum et al., 2017	5991 coronary CT angiography	Anomalous OCA from the opposite sinus (ACAOS)	129 patients with ≥1 ACAOS vessel with the following course subtypes: pre-pulmonic, subpulmonic, inter-arterial, retro aortic, and retrocardiac	5–83 years old	USA	68% men	Does not report
Li et al., 2016	22 transthoracic echocardiography	ALCAPA	22	12.9 ± 19.5 years old	Does not report	9 men and 13 women	Does not report
Grani et al., 2017	68 coronary computed tomography angiography (CCTA)	ACAOS	68	56 years old	Switzerland	73% men	Associated with adverse cardiac events in young people
Smettei et al., 2017	2235 computed tomography angiography (CTA)	Coronary artery abnormalities	241	24–77 years old	USA	166 men and 75 women	Syncope, chest pain, and sudden cardiac death
Temel et al., 2017	1138 catheterization and angiography	Anomalous of the coronary artery	42 were found. It was determined that 38 were anomalies of origin, 2 were anomalies of the intrinsic coronary artery anatomy, and 2 were anomalies of the coronary termination.	Does not report	Turkey	20 men and 22 women	Does not report
Dehaki et al., 2017	21	ALCAPA	21	22 days–51 years old	Iran	12 men and 9 women	Chest pain, dyspnea, and palpitations
Vinnakota et al., 2018	40 Transthoracic echocardiography, cardiac catheterization, computed tomography angiography, or cardiac magnetic resonance imaging	Anomalous aortic OCAs	40 were found. The coronary anomaly was from right to left in 35 patients, left to right in 4, and left coronary from the noncoronary sinus in 1 patient.	19–67 years old	USA	23 men and 17 women	Association with sudden cardiac death and symptoms similar to ischemia or arrhythmias
Nees et al., 2018	60	anomalous aorta of a coronary artery	60 were found; 30 with FTAA and 30 with ARCA	From 4 months to 68 years old	USA	38 men and 22 women	Chest pain and difficulty breathing
Driesen et al., 2018	30 cardiac catheterization	Anomalous coronary artery originating from the opposite sinus of Valsalva	25	36–64 years old	Does not report	19 men and 11 women	Chest pain
Türkoğlu et al., 2018	5165 coronary angiograms.	RCA originating from the left	35 were found (16 ACD originating from LCS, 13 LCx from the right coronaries (RCs) or ACD and 6 others). The most common form was RCA originating from LCS. Furthermore, we identified 5 cases of ACD originating in the LCS	17–90 years old	Does not report	24 men and 11 women	Does not report
Tian et al., 2018	110,158 coronary angiograms.	Anomalous OCAs	835 were found. The incidences of anomalous origin of the RCA, of the LCA, of both the RCA and the LCA, of the artery	Does not report	China	Does not report	Chest pain, dyspnea, palpitations, ventricular fibrillation, myocardial infarction, and even syncope, and sudden cardiac death
Yousif et al., 2019	39,577 angiographic studies	Anomalous of the coronary arteries	130 were found. The most prevalent anomaly overall was the LCx to RCA/sinus, which was present in *n* = 47 (36.2%).	Does not report	Switzerland	40 men and 90 women	Does not report
Finocchiaro et al., 2019	5100 sudden cardiac deaths	Anomalous OCA	30 were found. Anomalous ICA arising from the right sinus of Valsalva (ALCA) (*n* = 11) and anomalous ICA arising from the left sinus of Valsalva (ARCA) (*n* = 11) were the most common	16 years old	London	23 men and 7 women	Syncope and sudden cardiac death
Yakut et al., 2019	33 electrocardiogram (ECG), telecardiogram	ALCAPA	33	6–12 months	Turkey	11 men and 22 women	The most commonly presenting signs and symptoms were dyspnea, tachypnea, diaphoresis, prolonged feeding time, and developmental delay.
Sidhu et al., 2019	3233 coronary catheter angiograms	Coronary artery anomalies (CAAs)	99 were found. Split right coronary artery (RCA) was the most common coronary anomaly and was observed in 27 patients. Dual LADs were the second most common anomaly and were observed in 22 cases.	20–86 years old	India	74.75% men and 25.25% women	Acute coronary syndrome, stable IHD with exertional angina or exertional dyspnea, atypical chest pain with electrocardiographic (ECG) or echocardiographic changes, heart failure, or LV dysfunction
Al-Umairi et al., 2019	4445 coronary computed tomography angiography	Coronary abnormalities	59	12–80 years old	Sultanato de Omán	Does not report	Chest pain
Han et al., 2020	48,719 CT coronary angiography	left ACAOS	44 were found. The right sinus of Valsalva (RSV) was the most common origin (36/46, 78.26%).	Does not report	China	Does not report	Chest pain, palpitations, shortness of breath, and arrhythmia
Yu et al., 2020	30 echocardiographic examination	ALCAPA	24 ALCAPA	From 1 month to 51 years old	China	18 men and 12 women	Does not report
Ismail et al., 2020	29 echocardiographic	ALCAPA	29	Does not report	Saudi Arabia	15 men and 14 women	Does not report
Molosi et al., 2020	209	Anomalous aortic OCAy	163 were found; 116 anomalous right AC, 25 anomalous left AC, 17 single AC, and 5 anomalous circumflex AC	≤20 years old	USA	104 men and 53 women	Cardiac arrest/shock
Gaillard et al., 2020	61 anomalous aortic origin of a coronary artery	Anomalous aortic OCA	61 were found; 40 had right AAOCA and 21 had left AAOCA	Does not report	France	73.8% men	Chest pain
Bibevski et al., 2021	86 Echocardiograms	Anomalous origin of the RCA	86	16 years old	USA	52 men and 34 women	The presence of the variant can cause sudden death; in addition, given early diagnosis, surgical correction is suggested.
Pandey et al., 2021	955 multidetector CT angiography using dual-source scanner	Coronary artery abnormalities	79	2–5 years old	India	690 mn and 265 women	Does not report
Jiang et al., 2021	645 anomalous origin of a coronary artery	Anomalous OCA	167 were found. The RCA in 57% (96 of 167), the LCA in 23% (39 of 167), the LAD in 2% (4 of 167), LCx. in 16% (26 of 167), and multiple coronaries in 1% (2 of 167)	18 years and over	USA	91 men and 76 women	Chest pain and dyspnea
Kashyap et al., 2021	6258 coronary angiograms	Congenital coronary artery anomalies	129 were found. Anomalous origin and course of the coronary arteries were the most frequently observed anomalies (81 cases), followed by intrinsic anomalies of the coronary artery system in (44 cases).	32–81 years old	India	87 men and 42 women	Angina, dyspnea, syncope, acute coronary syndrome, heart failure, ventricular arrhythmias, and sudden cardiac death (SCD)
Cong et al., 2021	26 CTA images	RCA originating from the left sinus	26	62 years old	CHINA	17 men and 9 women	Dizziness and chest tightness.
Romeih et al., 2021	318 scanned with 128 dual-source multi-slice SOMATOM scanners (Siemens, Erlangen, Germany)	Anomalous OCA	20	From 1 month to 46 years old	Egypt	175 men and 143 women	Does not report
Ojha et al., 2021	21 arteriography	ALCAPA	21	From 2 months to 54 years old	India	8 men, 4 women, and 9 children	Gradually progressive dyspnea and growth retardation in children, asymptomatic adults
Jiang et al., 2022	118,167 patients from our cardiac catheterization database	Anomalous aortic OCA	793	Does not report	USA	Does not report	Does not report
Rodríguez et al., 2022	1486 computed tomography scanner	Anomalous coronary (AC)	70 were found. The coronary artery of the opposite coronary sinus was the most common (48.6%), with the RCA being the main anomalous artery (31%) and the main course being the interarterial (31%).	From 3 months to 90 years	Peru	4.3% women	Typical chest pain, dyspnea, palpitations, syncope, and electrocardiographic abnormalities
Wang et al., 2022	89	ALCAPA	89	Does not report	China	Does not report	Does not report
Xia et al., 2022	51	Anomalous origin of the LCA from the PA	51	12 months	China	22 men and 29 women	Does not report
Doan et al., 2023	220 computed tomography	Anomalous aortic origin of the RCA	220	<21 months	USA	60% men	Chest pain and exertional syncope
Lv et al., 2023	65	Anomalous OCA from the PA	65	<3 months	China	Does not report	Does not report
Yu et al., 2023	136	ALCAPA	136	1–53 years old	China	88 women and 48 men	Difficulty breathing and cough.

**Table 2:** Articles included for prevalence.

Author	Total, *n*	Prevalence
Harikrishnan et al., 2002	7400	34
Rodríguez et al., 2022	1486	70
Jiang et al., 2022	118,167	793
Romeih et al., 2021	318	20
Kashyap et al., 2021	6.258	129
Pandey et al., 2021	955	79
Han et al., 2020	48.719	44
Al-Umairi et al., 2019	4.445	59
Sidhu et al., 2019	3.233	99
Finocchiaro et al., 2019	5.100	30
Yousif et al., 2019	39.577	130
Tian et al., 2018	110.158	835
Türkoğlu et al., 2018	5.165	35
Temel et al., 2017	1.138	42
Smettei et al., 2017	2235	241
Gräni et al., 2016	5.634	145
Cheezum et al., 2017	5991	129
Krupińsk et al., 2015	7.115	62
Graidis et al., 2015	2572	60
Türkvatan et al., 2013	2.375	42
Akpinar et al., 2013	25.368	238
Opolski et al., 2013	8.522	72
Sivri et al., 2012	12.844	95
Xu et al., 2012	12.145	124
Morgan et al., 2012	1570	20
Rahman et al., 2012	6.065	79
Yang et al., 2010	6.014	66
Catanoso et al., 2010	6.300	23
Ugalde et al., 2010	10.000	121
Zhang et al., 2010	1.879	24
Cheng et al., 2010	3625	36
Yildiz et al., 2010	12.457	112
Fujimoto et al., 2010	5.869	89
Ouali et al., 2009	7.330	20
Eid et al., 2009	4.650	34
Tuncer et al., 2006	70.850	171
Durán et al., 2006	725	42
Schmitt et al., 2005	1758	28

## Supplementary Materials

The following supporting information can be downloaded at: https://www.mdpi.com/article/10.3390/diagnostics14131458/s1, Table S1: PRISMA 2020 Checklist. Reference [17] is cited in the Supplementary Materials.
